# Redox regulation of pyruvate kinase M2 by cysteine oxidation and S-nitrosation

**DOI:** 10.1042/BCJ20180556

**Published:** 2018-10-31

**Authors:** Alice Rose Mitchell, Meng Yuan, Hugh P. Morgan, Iain W. McNae, Elizabeth A. Blackburn, Thierry Le Bihan, Rafael A. Homem, Manda Yu, Gary J. Loake, Paul A. Michels, Martin A. Wear, Malcolm D. Walkinshaw

**Affiliations:** 1 Centre for Translational and Chemical Biology, School of Biological Sciences, University of Edinburgh, The King's Buildings, Max Born Crescent, Edinburgh EH9 3BF, U.K.; 2 Institute of Molecular Plant Sciences, School of Biological Sciences, University of Edinburgh, The King's Buildings, Max Born Crescent, Edinburgh EH9 3BF, U.K.

**Keywords:** enzyme activity, nitrosation, pyruvate kinase, redox signalling

## Abstract

We show here that the M2 isoform of human pyruvate kinase (M2PYK) is susceptible to nitrosation and oxidation, and that these modifications regulate enzyme activity by preventing the formation of the active tetrameric form. The biotin-switch assay carried out on M1 and M2 isoforms showed that M2PYK is sensitive to nitrosation and that Cys326 is highly susceptible to redox modification. Structural and enzymatic studies have been carried out on point mutants for three cysteine residues (Cys424, Cys358, and Cys326) to characterise their potential roles in redox regulation. Nine cysteines are conserved between M2PYK and M1PYK. Cys424 is the only cysteine unique to M2PYK. C424S, C424A, and C424L showed a moderate effect on enzyme activity with 80, 100, and 140% activity, respectively, compared with M2PYK. C358 had been previously identified from *in vivo* studies to be the favoured target for oxidation. Our characterised mutant showed that this mutation stabilises tetrameric M2PYK, suggesting that the *in vivo* resistance to oxidation for the Cys358Ser mutation is due to stabilisation of the tetrameric form of the enzyme. In contrast, the Cys326Ser mutant exists predominantly in monomeric form. A biotin-switch assay using this mutant also showed a significant reduction in biotinylation of M2PYK, confirming that this is a major target for nitrosation and probably oxidation. Our results show that the sensitivity of M2PYK to oxidation and nitrosation is regulated by its monomer–tetramer equilibrium. In the monomer state, residues (in particular C326) are exposed to oxidative modifications that prevent reformation of the active tetrameric form.

## Introduction

The Warburg effect is a frequent hallmark of cancer cells and describes the increased rate of glucose uptake and consequent aerobic glycolysis to produce ATP and lactate [1]. Proliferating cells eschew the standard oxidative phosphorylation pathway in favour of fermentation (even when oxygen is available), a process that may allow more efficient production of metabolite building blocks via glycolysis at the expense of more efficient ATP production in the mitochondria [2].

Pyruvate kinase (PYK) catalyses the final step of glycolysis and transfers a phosphoryl group from phosphoenolpyruvate (PEP) to ADP to produce pyruvate and ATP [3]. There are four pyruvate kinase isoforms in humans that are expressed in different tissue environments. Liver PYK (LPYK) is found in tissues where gluconeogenesis is carried out, primarily in the liver, but also in kidney and intestine [4]. Erythrocyte PYK (RPYK) is exclusively found in red blood cells and is very similar to LPYK, both enzymes being expressed from the same gene (*PKLR*) under the control of different tissue-specific promoters [5]. M1PYK and M2PYK are produced from the *PKM* gene by mutually exclusive alternative splicing; exons 9 and 10 are specific to M1- and M2PYK, respectively, while all other exons are common to both isoforms [6]. M1PYK is a highly active, non-allosteric isoform that is expressed in tissues where large amounts of ATP need to be produced rapidly, such as brain and muscle [7]. M2PYK is expressed in all proliferating cells and is a tightly controlled allosteric enzyme.

**Figure 1.**
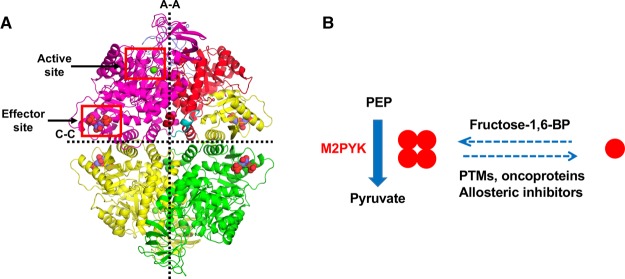
Allosteric regulation of M2PYK activity occurs through changing the tetramer–monomer equilibrium. (**A**) Architecture of the M2PYK tetramer. Chain A has been coloured to help to identify the different domains; N (cyan = residues 1–25), A (red = residues 25–116 and 220–402), B (blue = residues 117–219), and C (yellow = residues 403–531). The large (A–A) and small (C–C) interfaces are indicated by the dashed lines. The active site and effector site have been highlighted by red boxes. (**B**) M2PYK catalyses the final step of the glycolytic pathway, converting PEP to pyruvate and producing ATP. M2PYK exists in an oligomeric equilibrium between inactive monomer/dimer and active tetramer states. Activators, such as fructose-1,6-BP, promote the active tetramer state. Allosteric inhibitors, oncoproteins, and post-translational modifications (PTMs) have been found to promote the inactive monomer/dimer state.

The result of mutually exclusive splicing of the *PKM* gene transcript is that M1- and M2PYK differ in 22 residues within a 56 residue region situated at the C–C interface, where the C-domains of each monomer interact to form the PYK tetramer (Figure 1) close to the binding site for fructose 1,6-bisphosphate (F-1,6-BP), the allosteric activator of M2-, L-, and RPYK [6]. These differences alone allow M2PYK to exist in an allosterically regulated tetramer : dimer : monomer equilibrium while M1PYK is held in a constitutively active tetrameric state [8,9]. Comparison of the crystal structures of M1PYK and M2PYK bound to ATP, oxalate, and F-1,6-BP shows an overall r.m.s fit of ∼0.5 Å with the only significant difference being seen in the splice variant region [9].

M2PYK exists in a concentration-dependent tetramer : dimer : monomer equilibrium with a *K*_d_ for tetramer dissociation likely to be in the low micromolar range [9,10]. At sub-*K*_d_ concentrations, M2PYK dissociates into monomers that are inactive [9]. The tetramer dissociation rate is slow with a half-life of ∼15 min [10]. Small molecule allosteric activators and inhibitors have also been characterised: F-1,6-BP activates M2PYK by stabilising the tetrameric form of M2PYK in the R-state, while the inhibitory amino acids phenylalanine, tryptophan, and alanine bind in a different allosteric pocket and stabilise the inactive T-state tetramer [9,10].

M2PYK regulation is complex; its activity has been found to be influenced by more than 30 naturally occurring metabolites. Inhibitors include phenylalanine, thyroid hormone triiodo-l-threonine (T3), tryptophan, alanine, oxalic acid, and ribose 5-phosphate [9,10]. Phenylalanine and alanine have also been identified as inhibitors of M2PYK in rat and mouse [11–13]. In addition, tryptophan has been found to be an inhibitor of rat M2PYK [13]. In an extensive study of metabolite activators and inhibitors, no M1PYK activators were identified and only phenylalanine and oxalic acid were identified as weak inhibitors; in contrast, for M2PYK 14 activators were identified, with F-1,6-BP, histidine, and serine being the strongest [9,14]. F-1,6-BP has been identified as an allosteric activator of PYK in most organisms (e.g. *Escherichia coli* [15], globefish [16], and mammals [9]); however, exceptions include mycobacteria and *Toxoplasma* PYK which are activated by glucose 6-phosphate and AMP [17,18] and protist kinetoplastid PYK including that of the parasite *Leishmania* which is activated by fructose 2,6-bisphosphate (F-2,6-BP) [19].

Oxidation has also been implicated as a mechanism for inhibiting PYK orthologues including the yeast, *E. coli*, and human enzymes [20–22]. A study carried out using A549 human lung cancer cells found that an increase in intracellular reactive oxygen species (ROS) concentrations leads to decreased M2PYK activity [23]. This inhibition was reversed after the treatment of cell lysates with the reducing agent dithiothreitol (DTT). It was proposed that oxidation of M2PYK leads to dissociation of the homotetramer, a conclusion based on the observation that treatment of human lung cancer cells expressing FLAG-tagged M2PYK with the oxidising agent diamide prevented tagged M2PYK co-immunoprecipitating with endogenous M2PYK [23]. The significance of this for the cancer cells is explained by the authors in a model in which the regulation of M2PYK by the redox potential of the cell allows cancer cells to sustain antioxidant responses [23]. The idea is that inhibition of M2PYK would lead to accumulation of the glycolytic intermediate glucose 6-phosphate, which would then increase its entry into the pentose-phosphate pathway (PPP) thereby producing NADPH which is used by glutathione reductase (GR) to convert oxidised glutathione (GSSG) to reduced glutathione (GSH). This would have the effect of decreasing the oxidising potential of the cell and allowing oxidation of M2PYK to be reversed, leading to reactivation of the enzyme.

Here, we examine the effects of redox level on the oligomeric state and enzymatic activity of recombinant wild-type (WT) human M2PYK and many mutants with Cys-to-Ser mutations. We also show that S-nitrosation as well as oxidation regulate M2PYK oligomeric state and enzymatic activity. The evidence is also presented that stabilisation of the M2PYK tetramer can protect it from oxidation.

## Experimental

### Cloning of M2PYK mutants

Mutants of M2PYK were made using the QuikChange site-directed mutagenesis kit (Stratagene) using pET28a-M2PYK as a template and following the instructions in the manual. The following mutants were generated: C31A, C326S, C358A, C358S, C423S, C424A, C424S, and C424L. Mutations were confirmed by sequencing.

### Expression of human MPYKs in *E. coli*

The following procedure was used to express M2PYK WT, M1PYK WT, and all M2PYK mutants, except M2PYK C326S. Mutated plasmids were used to transform chemically competent *E. coli* BL21 cells (Novagen). Single colonies were used to inoculate 50 ml of LB media containing 50 µg/ml kanamycin and incubated at 37°C overnight with shaking. A total of 10 ml of cultures were taken from each of the overnight cultures and used to inoculate 1 l of 2xTY media containing 50 µg/ml kanamycin. In total, 1 l of cultures were grown to an OD_600_ of 0.8 at 37°C with shaking. Cultures were incubated at 4°C for 20 min before inducing expression by adding isopropyl-β-d-thiogalactopyranoside (IPTG) to a final concentration of 1 mM and incubating at 20°C for 18 h with shaking. Cells were harvested in a JLA-8.1000 rotor at 15 970×***g*** for 20 min at 10°C. Pellets from 1 l of cell cultures were flash frozen in liquid nitrogen before being stored at −80°C.

For expression of M2PYK C326S, the mutated plasmid was used to transform chemically competent *E. coli* BL21 star cells (Novagen). A single colony was used to inoculate 50 ml of LB medium containing 50 µg/ml kanamycin and incubated at 37°C overnight with shaking. Approximately 10 ml was taken from the overnight culture and used to inoculate 1 l of LB medium containing 50 µg/ml kanamycin. This 1 l culture was grown to an OD_600_ of 0.6–0.8 at 37°C with shaking. IPTG was added to a final concentration of 1 mM to induce expression and the culture was incubated at 18°C for 3 h with shaking. Cells were harvested in a JLA-8.1000 rotor at 15 970×***g*** for 20 min at 10°C. Cell pellets (1 l) were flash frozen in liquid nitrogen before being stored at −80°C.

### Preparation of cell lysates

All purification steps were carried out at 4°C unless otherwise stated. Cell pellets from 1 l of cell culture were resuspended in 30 ml of lysis buffer [50 mM NaH_2_PO_4_, 300 mM NaCl, 20 mM imidazole, pH 8.0, with EDTA-free protease inhibitors (Roche)]. Cells were lysed at 6°C by a single passage through a Constant Systems Cell Disruptor (1.1 kW TS Benchtop) set at 22 kpsi, followed by centrifugation at 58 500 ×***g*** for 45 min at 10°C, and then the supernatant was filtered through a 0.2 µm filter.

### Purification of bacterially expressed M PYKs

A clarified cell lysate was loaded onto a 5 ml IMAC HiTrap HP Sepharose column pre-charged with cobalt at 2 ml/min. The flow rate was maintained at 2 ml/min for the duration of the affinity purification step. The column was washed with 12 column volumes (CVs) of buffer A (50 mM NaH_2_PO_4_, 300 mM NaCl, 20 mM imidazole, pH 8.0) followed by an additional wash step with 15 CVs of 20% buffer B (50 mM NaH_2_PO_4_, 300 mM NaCl, 250 mM imidazole, pH 8.0) to wash off contaminating proteins. The His-tagged MPYK protein was eluted using a 5 CV step gradient of 100% buffer B. Eluted fractions were pooled and concentrated with a Vivaspin concentrator (molecular mass cut-off 30 kDa) before loading onto a HiLoad 16/600 Superdex 200 column gel filtration column pre-equilibrated with Dulbecco's phosphate-buffered saline without calcium and magnesium (PBS-CM — Sigma Cat. No. D5652). A flow rate of 1 ml/min was used throughout the gel filtration process. Eluted fractions were pooled and concentrated to ∼20 mg/ml using a Vivaspin column with a molecular mass cut-off of 30 kDa.

### Measurement of protein concentration

Protein concentration was determined by measuring absorbance at 280 nm and the molar absorption coefficient *ε*_280 nm_ =  28 600 M^−1^ cm^−1^. Concentrated samples in PBS-CM were flash frozen with liquid nitrogen and stored at −80°C.

### Measuring PYK activity using the lactate dehydrogenase-coupled assay

Protein solutions (5 ml) of 0.0025 mg/ml M PYK were prepared by dilution in PBS-CM (pH 7.4) with or without the addition of additives. The protein solutions were incubated at room temperature for 3 h to ensure oligomeric equilibrium was reached. Assay buffer comprised PBS-CM, 20 mM MgCl_2_, 200 mM KCl, 4 mM ADP, 1 mM NADH, and 30 U/ml LDH (lactate dehydrogenase). Approximately 5 ml of 2× assay buffer was taken and PEP added to a concentration of 10 mM. The final mixture was re-adjusted to pH 7.4 after the addition of compounds.

Two-fold serial dilutions of PEP were prepared in a 96-well master block (Greiner Bio-one Cat. No. 780270) using 2× assay buffer for dilution yielding PEP concentrations ranging from 78 µM to 10 mM (final assay concentrations 39 µM to 5 mM). A multi-channel pipette was used to transfer 50 µl of each PEP dilution to a column of a 96-well plate (Greiner Bio-one Cat. No. 655101).

The reaction was started by adding 50 µl of protein solution to the PEP titrations in the 96-well plate using a multi-channel pipette. Final concentrations of reagents were as follows: 0.00125 mg/ml M PYK, 10 mM MgCl_2_, 100 mM KCl, 2 mM ADP, 15 U/ml LDH, 0.5 mM NADH, and PEP concentrations ranging from 39 µM to 5 mM.

The assay solutions and protein solutions were pre-incubated at 37°C for 10 min before mixing together. Plates were agitated for 10 s before measuring the decrease in absorbance at 340 nm for 5 min using a plate reader which was set at 37°C (SpectraMax M5 multimode plate reader, Molecular Devices). Initial reaction rates were obtained using the SoftMax Pro software. Substrate-velocity graphs were plotted using Kaleidograph (Synergy Software).

### Measuring PYK activity using the Kinase Glo assay

Protein solutions (5 ml) of 0.0025 mg/ml M1PYK or M2PYK were prepared by diluting in PBS-CM and were incubated at room temperature for 3 h with either 10 µM H_2_O_2_ or with 1 mM DTT to ensure oligomeric equilibrium was reached. Assay buffer comprised: PBS-CM, 20 mM MgCl_2_, 200 mM KCl, and 4 mM ADP. About 5 ml of 2× assay buffer was taken and PEP added to a concentration of 2 mM and then transferred to a 96-well plate (Greiner Bio-one Cat. No. 655101). The reaction was started by adding 25 µl of protein solution to the PEP solution in the 96-well plate using a multi-channel pipette. The final concentrations of the reagents were as follows: 0.00125 mg/ml M PYK, 10 mM MgCl_2_, 100 mM KCl, 2 mM ADP, and 1 mM PEP. The reaction was incubated for 30 min before adding 50 µl of Kinase Glo Plus reagent (Promega) and incubated at room temperature in the dark for 10 min. Endpoint luminescence readings were measured using a SpectraMax M5 multimode plate reader at room temperature.

### Dynamic light scattering

Mass distribution analysis of M2PYK mutant protein solutions by dynamic light scattering was performed on a Zetasizer APS (Malvern Instruments) with five repeat runs of 60 μl (1 mg ml^−1^) in PBS-CM, at 22°C, with a 120-s equilibration.

### Thermal shift assay

The SYPRO Orange dye is supplied by Invitrogen (catalogue no. S6650) at 5000× concentration in DMSO and was diluted in PBS-CM before use. Test samples were prepared in a 96-well PCR plate (Bio-Rad) before adding SYPRO Orange dye and sealing the plate with optical quality tape (Bio-Rad). Each 50 µl sample contained 0.55 mg/ml protein in PBS-CM with 1.11 mM F-1,6-BP, 1 mM H_2_O_2_, or 1 mM DTT. The 96-well plate was heated in an i-Cycler iQ5 real-time PCR detection system (Bio-Rad) from 20 to 80°C in increments of 1°C. A charge-coupled (CCD) camera was used to monitor the fluorescence changes in the wells simultaneously using an excitation wavelength of 485 nm and an emission wavelength of 575 nm.

### Analytical size-exclusion chromatography

Analytical size-exclusion chromatography was carried out as described previously [9]. M1- and M2-PYK protein samples were incubated overnight at room temperature at 0.1 mg/ml before loading independently onto a calibrated Superdex 200 PC 3.2/30 gel filtration column (GE Healthcare), with a total bed volume of 2.38 ml, pre-equilibrated in PBS-CM at room temperature, and run at 0.1 mg/ml. To ensure loading consistency between runs, a 25 µl loop was filled with 50 µl of the sample, followed by a run load-volume of 50 µl. UV absorbance was monitored at both 280 and 214 nm, with a 10 mm path length flow-cell.

### *In vitro* biotin-switch assay

*In vitro* biotin-switch assay was carried out for the identification of S-nitrosated proteins. Protein samples (pre-incubated with or without 1 mM F-1,6-BP or 4 mM phenylalanine) were prepared by diluting to a concentration of 0.17 mg/ml in HEN buffer (250 mM HEPES, 1 mM EDTA, 0.1 mM neocuproine, pH 7.7). CysNO was used as the NO donor and was prepared by mixing 20 µl of 100 mM l-cysteine (dissolved in 200 mM HCl) with 20 µl of 100 mM NaNO_2_ (dissolved in H_2_O) resulting in 50 mM solution of CysNO, which was then added to protein samples to a final concentration of 1 mM for protein S-nitrosation. The reaction was incubated in the dark for 20 min at room temperature. The addition of 0.1% SDS for exposing all cysteine residues was used as a positive control. No CysNO was added for the negative control sample.

After incubation, CysNO donor was removed by passing the samples through pre-equilibrated Zeba-spin columns. NEM (*N*-ethylmaleimide)-blocking buffer (HEN buffer, 5% SDS, and 50 mM NEM) was added to unfold the protein and block all unmodified cysteines, and the reaction was incubated at 50°C for 30 min. Two volumes of −20°C 100% acetone were then added and the samples were incubated at −20°C for 20 min. Precipitated protein was pelleted by centrifuging at 14 000×***g*** for 5 min at 4°C. The supernatant was removed. The protein pellet was washed three times with −20°C 70% acetone. Residual acetone was allowed to evaporate by placing tubes uncapped in the dark at room temperature.

Protein pellets were resuspended in 85 µl of HENS buffer (HEN buffer with 1% SDS). Biotin-labelling solution (final concentration: 25 mM ascorbate and 0.4 mM biotin-HPDP) was added and the samples were incubated for 1 h at room temperature. In this step, any nitrosated cysteine can be reduced by ascorbate and thus biotinylated by biotin-HPDP. Non-nitrosatable cysteines were blocked by NEM and thus not biotinylated. After biotinylation, proteins were collected by acetone precipitation again: two volumes of −20°C 100% acetone were then added and the samples were incubated at −20°C for 20 min. Precipitated protein was pelleted by centrifuging at 14 000×***g*** for 5 min at 4°C. The supernatant was removed. The protein pellet was washed for three times with −20°C 70% acetone. Residual acetone allowed to evaporate by placing tubes uncapped in dark at room temperature.

Each protein pellet sample was resuspended with 300 µl HENS buffer (25 mM HEPES, 1 mM EDTA, 1 mM neocuproine, and 1% SDS). Twenty microlitres per each sample were taken out as an ‘input’ control. For each of the rest 280 µl sample, 1.5 ml of neutralisation buffer (25 mM HEPES, 100 mM NaCl, 1 mM EDTA, and 0.5% Triton X-100, pH 7.5) and 20 µl of streptavidin beads were added to pull-down the biotinylated (i.e. S-nitrosated) proteins.

After an overnight incubation at 4°C, streptavidin beads conjugated with biotinylated proteins were centrifuged down 30 s at 2200 ×***g***. Unbound proteins were removed by five times washing with neutralisation buffer. Biotinylated samples were eluted using buffer containing 1% β-mercaptoethanol (each sample was incubated with 20μl of elution buffer for 30 min at room temperature).

After an SDS–PAGE was run for each eluted sample, the protein bands were transferred onto a nitrocellulose membrane at 80 V at 4°C for 90 min for further western blot analysis. The membrane was blocked with PBST (PBS with 0.05% Tween 20) supplemented with 5% skimmed milk powder at room temperature for 1 h. The blocked membrane was then incubated with 1 : 2000 diluted HRP-linked anti-His-tag antibody overnight at 4°C. After the membrane was washed three times with PBST with 5% skimmed milk powder followed by twice with PBS, ECL solutions were applied for luminescence exposure.

### Crystallisation screening procedures

Purified M2PYK C358A, M2PYK C358S, and M2PYK C424A samples that had been stored at −80°C were used for the experiments. Proteins were thawed on ice before mixing with ligands. Crystallisation experiments were carried out by the vapour diffusion method and the hanging drop technique at 18°C (M2PYK C424A) or 4°C (M2PYK C358S).

Crystallisation drops were set up by mixing 1.5 µl of well solution with 1.5 µl of 10 mg/ml protein sample containing 5 mM of each ligand [9]. The well solution consisted of 6–16% PEG 3350, 100 mM sodium cacodylate (pH 6), 20 mM triethanolamine–HCl buffer (pH 7.2), 50 mM MgCl_2_, and 100 mM KCl. The drops were equilibrated against 1 ml of well solution.

### Data collection and processing

X-ray intensity data from crystals were collected at the Diamond synchrotron radiation facility in Oxfordshire, U.K. on beamline IO2. Prior to data collection, crystals were equilibrated over a well solution containing a high concentration of PEG-3350 to help dehydrate the crystal and eliminate the appearance of ice-rings. The intensity data were collected from single crystals flash frozen in liquid nitrogen at 100 K. Processing was carried out by the Xia2 automated data reduction system.

### Model building and refinement

The M2PYK C358S structure was solved by molecular replacement using MolRep [24] using the AC domains of the M2PYK structure with PDB ID: 3SRH. Because the B-domains of the M2PYK C358S structure have very poor density, they were solved separately. One of the B-domains was found by using a B-domain of M2PYK 3SRH separately in MolRep refinement. The other three B-domains were placed by finding the corresponding B-domains in the rabbit M2PYK structure with PDB ID: 1PKN. The structure was then refined using REFMAC5 [25]. The M2PYK C424A structure was solved by molecular replacement using MolRep. The M2PYK structure with PDB ID: 3SRD was used as the search model because the two crystals are isomorphous. The structure was then refined using REFMAC5.

## Results and discussion

### Redox level regulates the stability, oligomeric state, and enzymatic activity of M2PYK

#### Oxidising conditions favour monomeric M2PYK

**Figure 2.**
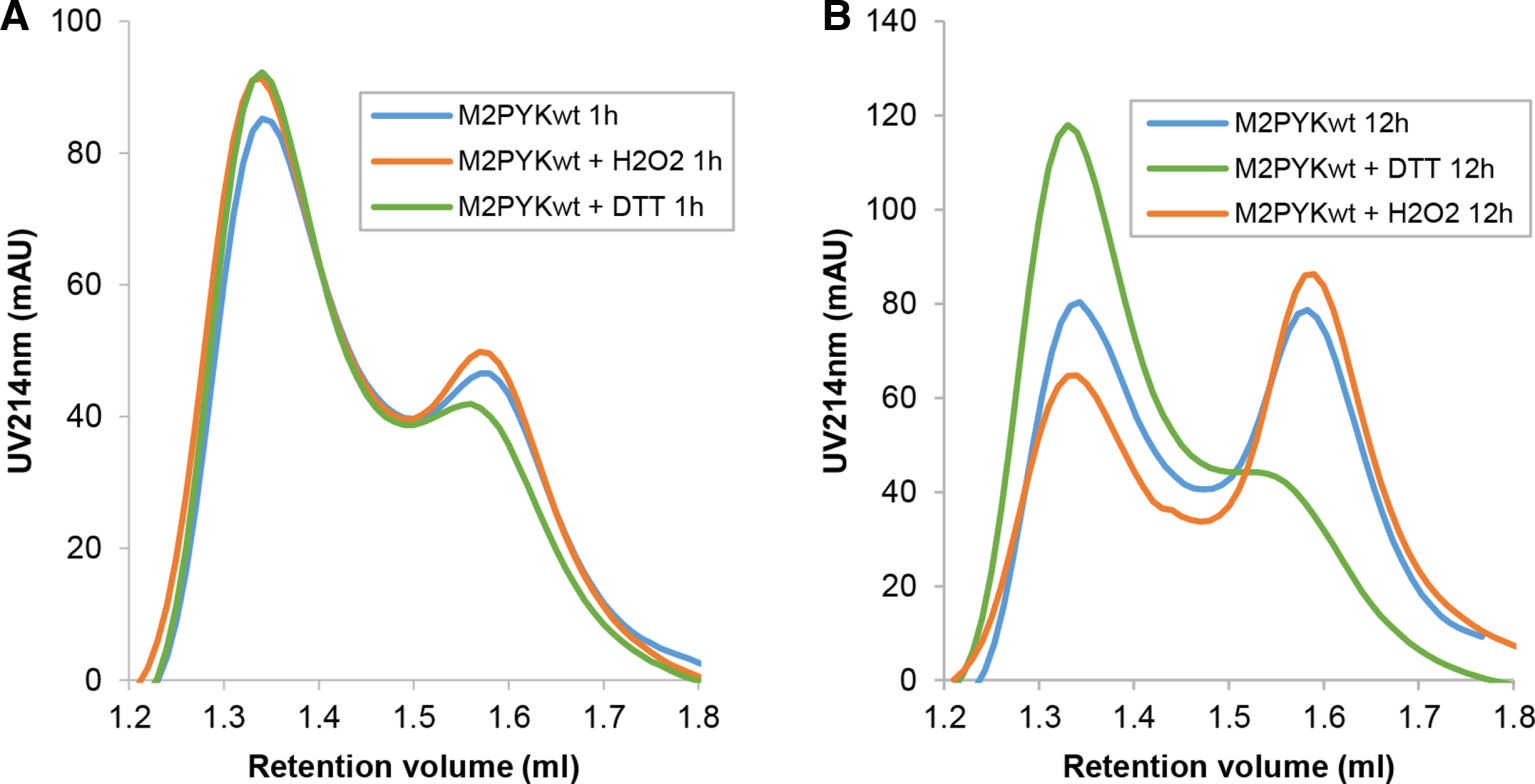
Effects of redox environments on the oligomerisation of M2PYK. M2PYK stock solution of ∼20 mg/ml was diluted to 0.1 mg/ml in PBS-CM and incubated, in the absence and presence of 1 mM DTT or 20 μM H_2_O_2_, at room temperature for (**A**) 1 h and (**B**) 12 h before injecting a 25 µl sample into a Superdex® 200 PC 3.2/30 gel filtration column pre-equilibrated in PBS-CM. Assuming a bimodal tetramer (T): monomer (M) distribution, the peak areas correspond to:$$\begin{array}{rl} & \text{M2PYK}\;(-\text{DTT},\;-\;{\text{H}}_{2}{\text{O}}_{2})\;\text{at}\;1\;\text{h}\;(62\%\;\text{T},\;38\%\;\text{M})\;\text{and}\;\text{at}\;12\;\text{h}\;(55\%\;\text{T},\;45\%\;\text{M})\\ & \text{M2PYK}\;(-\text{DTT},+\;{\text{H}}_{2}{\text{O}}_{2})\;\text{at}\;1\;\text{h}\;(63\%\;\text{T},\;37\%\;\text{M})\;\text{and}\;\text{at}\;12\;\text{h}\;(42\%\;\text{T},\;58\%\;\text{M})\\ & \text{M2PYK}\;(+\text{DTT},-\;{\text{H}}_{2}{\text{O}}_{2})\;\text{at}\;1\;\text{h}\;(64\%\;\text{T},\;36\%\;\text{M})\;\text{and}\;\text{at}\;12\;\text{h}\;(71\%\;\text{T},\;29\%\;\text{M}). \end{array}$$

Analytical gel filtration was used to investigate the effect of oxidation on the oligomeric state of M2PYK. A stock solution of M2PYK (∼20 mg/ml) was diluted to 0.1 mg/ml in PBS-CM and then incubated in the presence and absence of DTT or H_2_O_2_ at room temperature for 1 h (Figure 2A) and 12 h (Figure 2B) before injecting onto a Superdex 200 PC 3.2/3.0 gel filtration column pre-equilibrated in PBS-CM. The two major peaks run with identical elution volumes as those previously identified using size-exclusion multi-angle scattering as tetramers and monomers with absolute molar masses of 214 and 53 kDa, respectively [9]. A series of analytical gel filtration runs have been used to show that M2PYK dissociates over time until an equilibrium between tetramer and monomer is reached (Figure 2, blue lines). The tetramer–monomer dissociation *K*_d_ is estimated to be ∼0.9 μM with an apparent dissociation rate with *t*^1/2^  ∼ 15 min [10]. After incubation at the lower concentration for 1 h [following dilution from ∼350 µM (20 mg/ml) to ∼1.7 µM (0.1 mg/ml)], a small effect on the monomer tetramer distribution can be observed (Figure 2A). The difference after 12 h once equilibrium had been reached is marked: DTT maintained the majority of M2PYK as tetramers, whereas incubation with H_2_O_2_ clearly shifted the equilibrium towards a higher proportion of monomers (Figure 2B). These experiments are therefore consistent with the conclusion that a reducing environment promotes tetramerisation of M2PYK, while oxidation promotes dissociation. The similarity in Figure 2 between control M2PYK (measured under ambient conditions) and the H_2_O_2_-treated enzyme suggests that the oxidising effects of air also significantly affect the M2PYK oligomeric state.

#### Oxidising conditions decrease the thermostability of M2PYK

**Figure 3.**
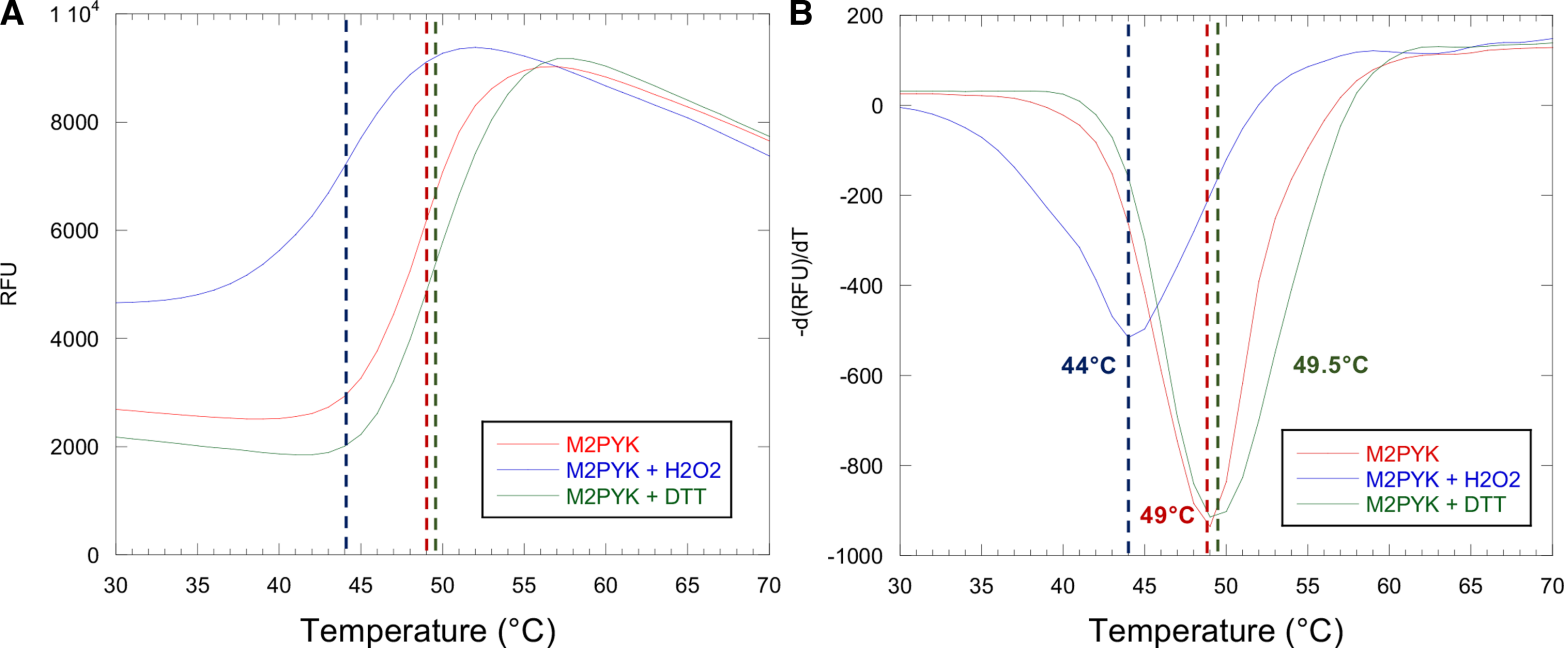
Thermal stability of M2PYK and the effects of redox environments. Thermal denaturation data for M2PYK (final concentration 0.49 mg/ml) in the presence of 1 mM DTT (green), 1 mM H_2_O_2_ (blue), or with no additives (red). RFU, relative fluorescence units; −d(RFU)/dT, negative derivative of RFU.

To determine the effect of oxidising conditions on the stability of M2PYK, a thermal shift assay was carried out in the presence of 1 mM DTT or 1 mM H_2_O_2_. DTT (1 mM) or H_2_O_2_ was added to 0.55 mg/ml M2PYK before the addition of the SYPRO Orange environmentally sensitive dye and measurement of fluorescence using a real-time PCR detection system as it heated the samples in increments of 1°C from 20 to 80°C (Figure 3).

Incubation of M2PYK with DTT resulted in a slight increase in melting temperature (Figure 3), indicating that the reducing conditions stabilise the structure of M2PYK, but not to the extent as achieved by the allosteric activator F-1,6-BP which increases the melting temperature by ∼7°C [9]. In contrast, the presence of H_2_O_2_ causes a significant decrease in *T*_m_ of 5°C, showing that oxidising conditions have a destabilising effect on M2PYK structure (Figure 3). Thermostability of M1PYK is not affected by either oxidising or reducing conditions, thus showing that the observed effect on M2PYK is not simply a non-specific effect of redox conditions on protein structure.

#### Oxidising conditions decrease the enzymatic activity of M2PYK

**Figure 4.**
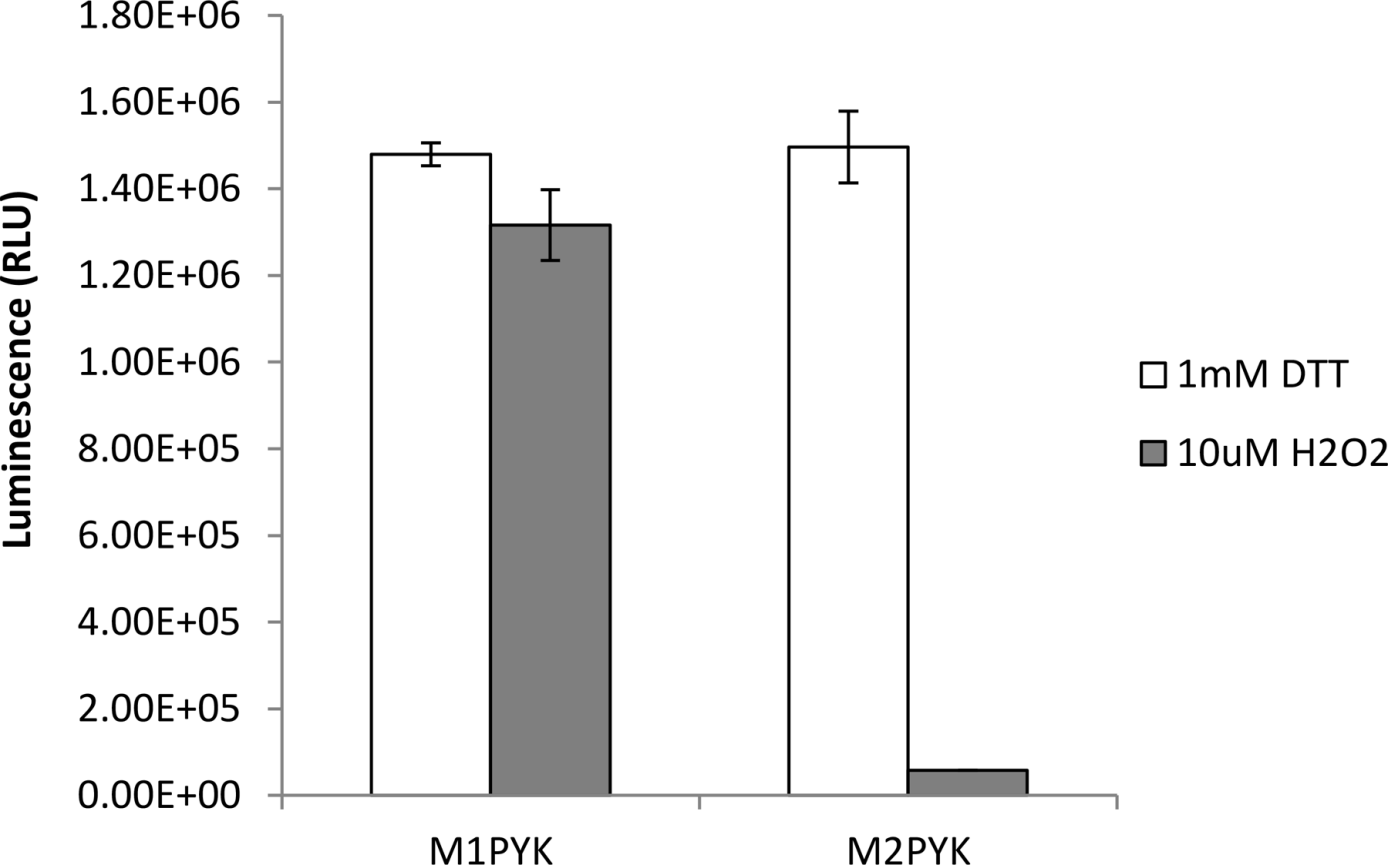
The enzymatic activity of M2PYK is significantly decreased in oxidising conditions. The Kinase Glo assay was used to measure the activity of M1PYK and M2PYK (0.0025 mg/ml) in the presence of 1 mM DTT (reducing conditions) or 10 µM H_2_O_2_ (oxidising conditions).

The activity of M2PYK in the presence of 1 mM DTT or 10 µM H_2_O_2_ was measured in order to investigate the effect of oxidising conditions on its activity. As a control, the activity of the constitutively active and tetrameric M1PYK was also measured. Before measuring the activity using the Kinase Glo assay kit, the M1PYK and M2PYK proteins (0.0025 mg/ml) were incubated at room temperature for 3 h in order to allow oligomeric equilibrium to be reached. The results show that oxidising conditions significantly decrease the activity of M2PYK when compared with reducing conditions, whereas there is little change in the activity of M1PYK (Figure 4). The observation that M1PYK activity is unaffected by oxidising conditions provides additional support to the hypothesis that the effect on M2PYK is specific, especially given the similarity in sequence and overall structure between the two isoforms.

#### Reducing agents increase the enzymatic activity of M2PYK

**Figure 5.**
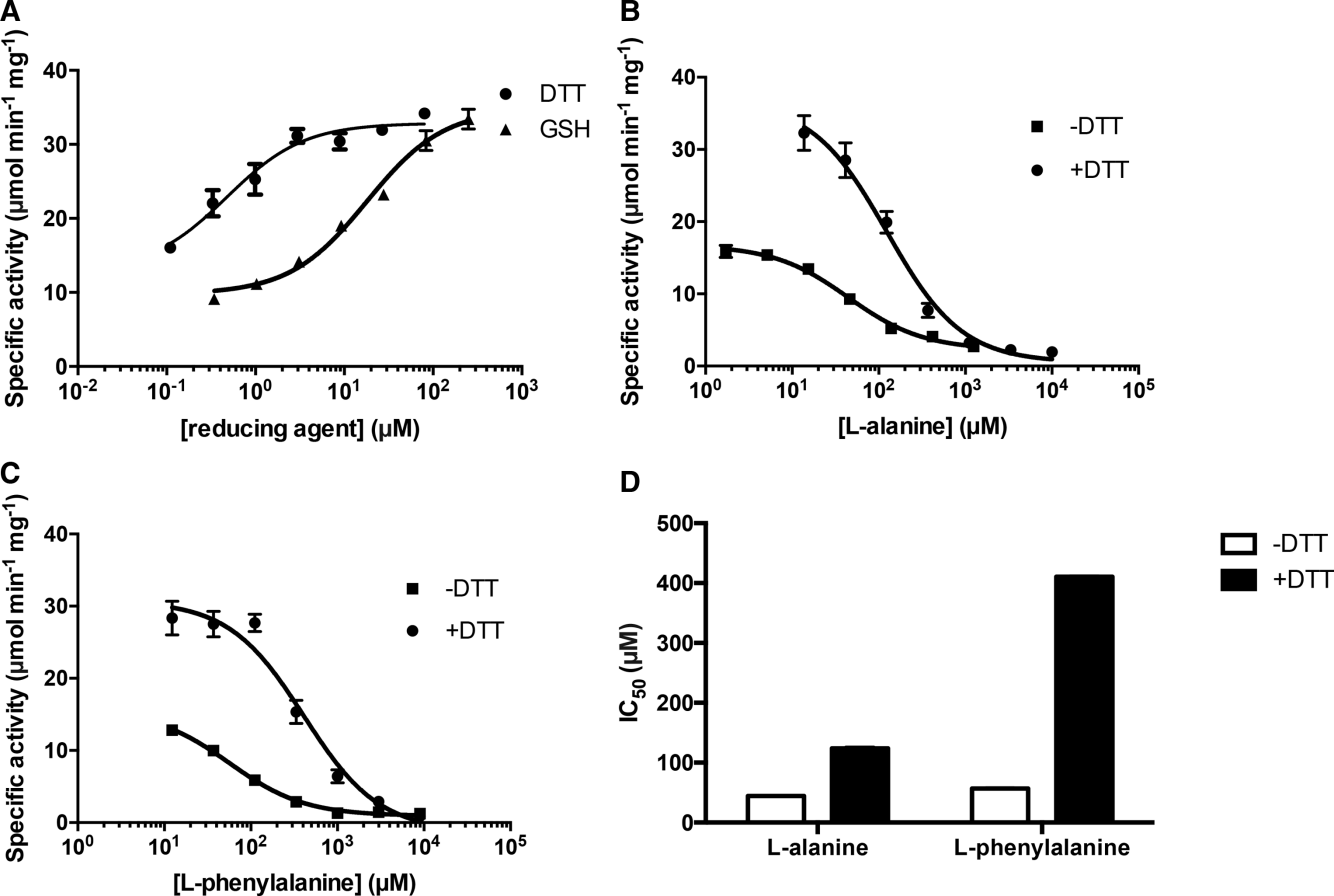
Reducing agent affects the enzymatic activity of M2PYK and its sensitivity to amino acid inhibitors. (**A**) Reducing agents increased the enzymatic activity of M2PYK. The titrations of DTT and GSH showed that the enzymatic activity of M2PYK is redox-dependent. (**B**) The inhibitory effect of l-alanine on the enzymatic activity of M2PYK was regulated by DTT. (**C**) The inhibitory effect of l-phenylalanine on the enzymatic activity of M2PYK was regulated by DTT. (**D**) The presence of DTT desensitised M2PYK to the inhibitory effects of free amino acids. The IC_50_ values of amino acid inhibitors were significantly increased by the addition of DTT (present in **B**, **C**, and **D** at a concentration of 100 µM). Data represent the mean ± SEM of three independent experiments.

The effect of DTT and GSH on the enzymatic activity of M2PYK is shown in Figure 5A. At saturating conditions (∼ 100 µM), both reducing agents increase the specific activity by ∼4-fold. The fact that both GSH and DTT showed activating effects suggests that activation is due to the reducing properties of DTT and GSH rather than a specific ligand-binding effect.

We have previously shown that M2PYK acts as an amino acid nutrient sensor and is inhibited by Ala, Phe, and Trp and activated by Ser [10]. Here, we show that this allosteric regulatory mechanism is further modulated by oxidising or reducing conditions. IC_50_ values of l-alanine and l-phenylalanine increased by up to 10-fold in the presence of DTT (Figure 5B–D).

**Figure 6.**
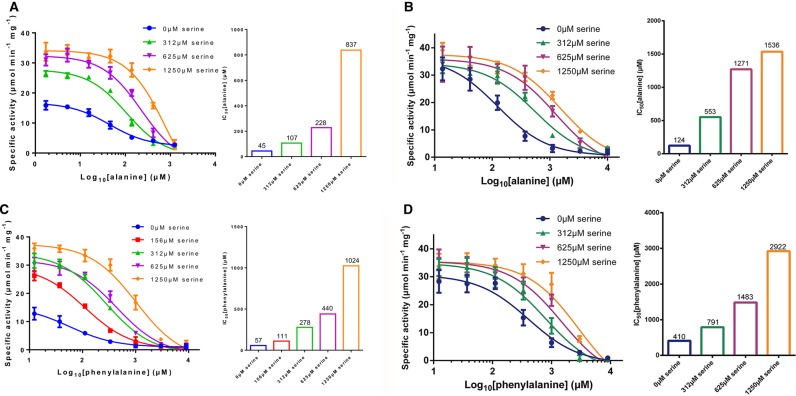
Competing and complementary effects of activator (Ser) and inhibitors (Ala, Phe) in the presence and absence of DTT. (**A**) (left) Inhibitory effect of Ala in the presence of different concentrations of the activator Ser. (Right) bar chart shows how the IC_50_ for Ala increases in the presence of increasing concentrations of Ser. (**B**) Same as (**A**) but in the presence of 1 mM DTT which significantly reduces the affinity for Ala. (**C**) Same as (**A**) but with Phe rather than Ala. (**D**) Same as (**C**) but in the presence of 1 mM DTT which significantly reduces the affinity for Phe.

Figure 6A,B shows the combined effects of the inhibitor Ala with the activator Ser in the presence and absence of the reducing agent DTT. The amino acids compete for the same allosteric site, so that the relative concentrations of inhibitor/activator govern the degree to which M2PYK is activated [10]. Under reducing conditions, the effect of the Ala inhibitor is lessened with an IC_50_ ∼124 µM compared with 45 µM with no DTT present. Increasing Ser concentration further reduces the inhibitory effect of Ala which is expected as Ala and Ser compete for the same allosteric site. A similar effect is observed for Phe and Ser (Figure 6C,D), where the IC_50_ for the inhibitor Phe is increased from 57 to 410 µM in the presence of 1 mM DTT. As with Ala, there is an increase in the IC_50_ of Phe as the Ser concentration increases. These IC_50_ changes show that the activation of M2PYK under reducing conditions complements the binding of the activating Ser but antagonises binding of the inhibitory amino acids Phe and Ala. X-ray structures of active R-state (Ser-bound) M2PYK tetramers have a different conformation to the Phe/Ala-bound T-state [9,10]. The activity results are therefore consistent with reducing agents favouring an R-state conformation which disfavours Ala or Phe fitting in the allosteric amino acid-binding pocket.

### The role of Cys residues in M2PYK redox regulation

**Figure 7.**
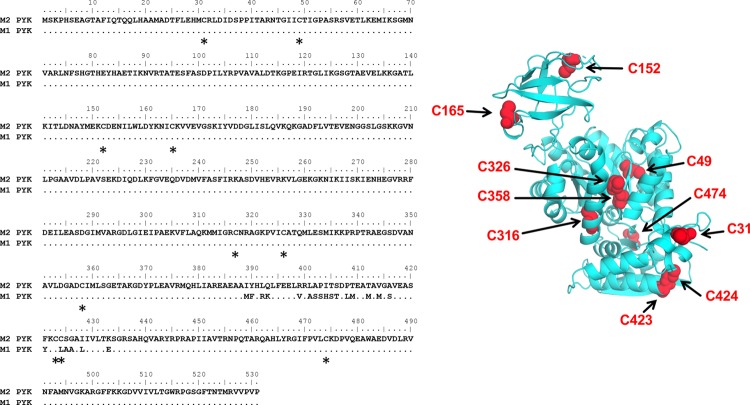
Positions of cysteines in M2PYK sequence and structure. Left, sequence alignment of M1 and M2PYK showing positions of 10 cysteines (indicated by asterisks) and the 22 residues that differ between M1 and M2PYK within the splice variant region. Right, a cartoon of M2PYK monomer showing the positions of cysteines in the folded structure.

Ten cysteines are found in the sequence of M2PYK (i.e. 1.9% of the amino acid composition) (Figure 7). A cysteine content of 1.9% is about average for a mammalian intracellular protein, which is typically 2.2% [26]. Many of the cysteines of M2PYK are readily accessible to solvent and thus modification by oxidising agents, suggesting that regulation of M2PYK by oxidation may be complex and involve multiple cysteines.

#### Effect of mutations on the C–C interface: C424S, C424A, and C424L

M2PYK C424 is the only cysteine unique to M2PYK, with the equivalent residue in M1PYK being a leucine. This cysteine is situated within the splice variant region at the C–C interface, where the C-domains of each monomer interact to form the PYK tetramer. The splice variant region encodes residues 378–434, and within this region, only 22 amino acids differ between the constitutively active M1PYK and the allosteric M2PYK (Figure 7). We have made point mutations in M2PYK by site-directed mutagenesis to create M2PYK C424S, M2PYK C424A, and M2PYK C424L.

A thermal shift assay was carried out to compare the thermal stability of the M2PYK C424 point mutants with that of M1PYK and M2PYK WT enzymes in oxidising and reducing conditions. Mutants in which C424 has been replaced with a hydrophobic residue, C424A and C424L, have very similar thermal stability as M2PYK WT (data not shown). The hydrophilic mutation C424S, however, results in a decrease in thermal stability and a higher sensitivity to treatment with H_2_O_2_ (Figure 3) [27]. A decrease in stability with an increasing hydrophilicity of cysteine-point mutants may explain the destabilisation of M2PYK in oxidising conditions where hydrophilic sulfonate or sulfinate adducts are likely to be generated [28].

**Figure 8.**
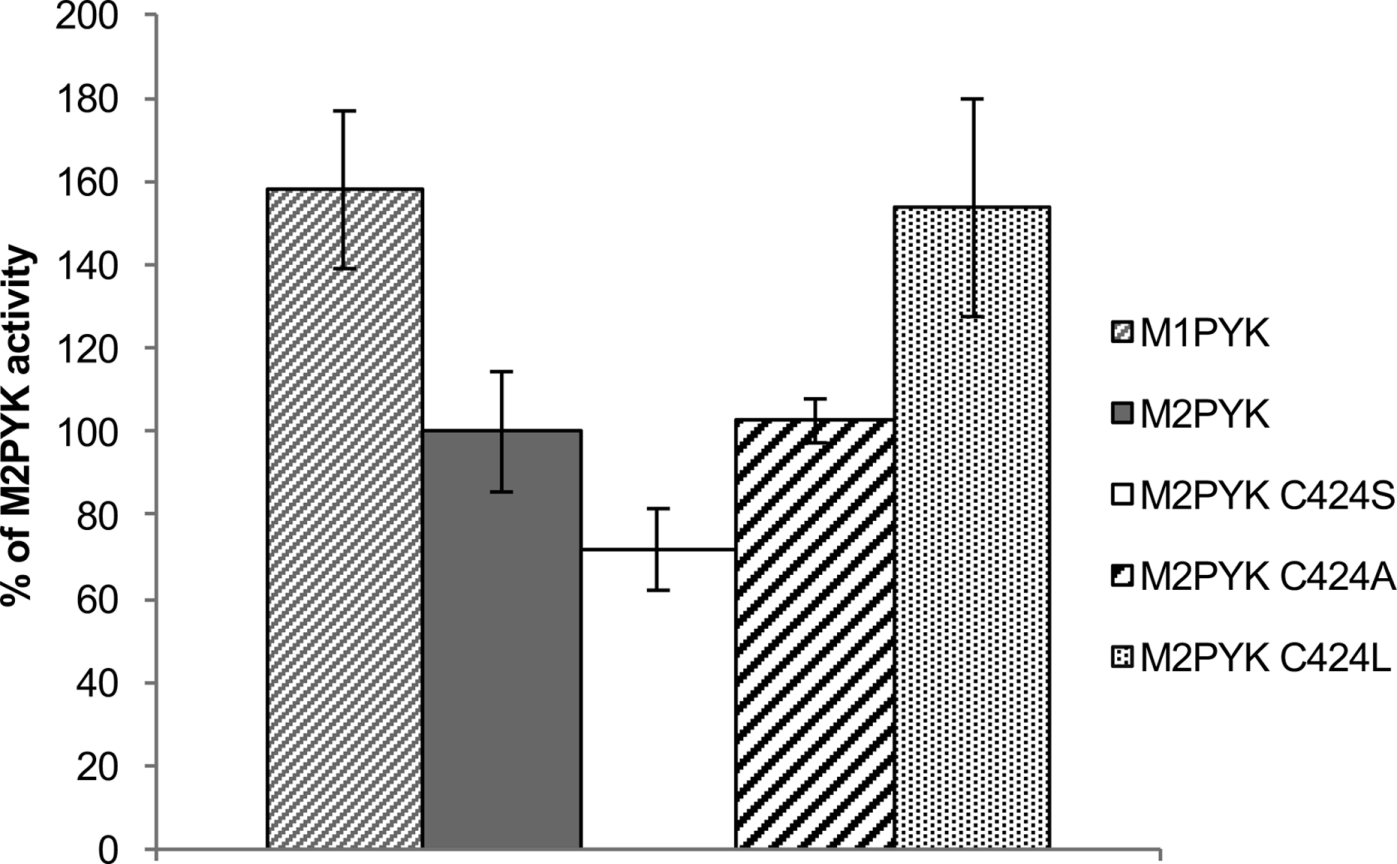
Increasing the hydrophobicity of residue 424 increases M2PYK enzymatic activity. Enzymatic activity was measured using the LDH-coupled assay with 0.00125 mg/ml M2PYK in the presence of 1 mM DTT and saturating substrate concentrations (1 mM PEP and 2 mM ADP). Velocity values (in Abs units/s) were calculated and are presented here as a percentage of the M2PYK WT velocity**.**

Enzymatic activities of M2PYKC424S, M2PYKC424A, and M2PYKC424L were measured using the LDH-coupled spectrophotometric assay. A summary of kinetic data for site point mutants and WT enzymes is given in Supplementary Table S1. Velocity (absorbance units/s) values are presented in Figure 8 as percentages of the M2PYK WT velocity. The activity of M1PYK was also measured as Cys424 is the only cysteine of M2PYK that is not present in M1PYK. The results show that as the hydrophobicity by the mutation is increased, the activity of the enzyme also increases, with M2PYK C424L having a similar activity to M1PYK in the absence of the allosteric activator F-1,6-BP (Figure 8). These results fit well with the thermal shift data, showing that the less stable C424S mutant has a lower activity than M2PYK WT, while C424A has very similar thermal stability and enzyme activity to WT and the C424L mutant is the most stable and is most active. These results are in agreement with the results of Ikeda and Noguchi who found that the C424L mutation in rat M2PYK resulted in increased activity and loss of allosteric regulation by F-1,6-BP [29].

M2PYK C424A was co-crystallised with F-1,6-BP, oxalate, and ATP and crystals diffracted to a maximum resolution of 2.87 Å. A model of M2PYK WT (PDB code: 3SRD) was used to calculate the |*F*_o _− *F*_c_| difference electron density map with M2PYK C424A. The difference electron density map gave a clear negative peak where the cysteine has been substituted by alanine consistent with the C424A mutation (Supplementary Figure S2). Superposition of the M2PYK C424A structure with that of the M2PYK WT holo structure (PDB code: 3SRD) results in an overall RMS deviation of 0.57 Å. The maximum displacement between M2PYK WT holo and M2PYK C424A is 3 Å which occurs at around residue 126 on each of the four chains. This difference is caused by the B-the domain of the M2PYK C424A structure adopting a slightly more closed orientation than in the M2PYK WT structure.

#### Characterisation of the C326S and C358S mutants

Cys326 is located at the A–A interface and is therefore completely buried in tetrameric M2PYK. However, in the monomeric form, Cys326 is exposed to the solvent making it accessible to oxidising agents (Figure 1). Cys358 is situated in the β-barrel close to the active site and its buried location makes it a surprising candidate for modification. It has, however, previously been identified as being important in the regulation of M2PYK by oxidation [23]. We have made point mutations in M2PYK by site-directed mutagenesis to create M2PYK C326S and M2PYK C358S.

It has been inferred from cellular studies that the enzymatic activity and the oligomeric state of M2PYK depend on its redox state [23]. The suggestion that Cys358 was the main residue that was affected by oxidation was based on the observation that the activity of the mutant M2PYK C358S is free from effects of reducing agents or oxidants. However, as we have shown with the C424 mutants, the hydrophobicity of the side chain can also have an effect on tetramer stability and factors other than oxidation of Cys358 may play a role in the observed loss of activity.

**Figure 9.**
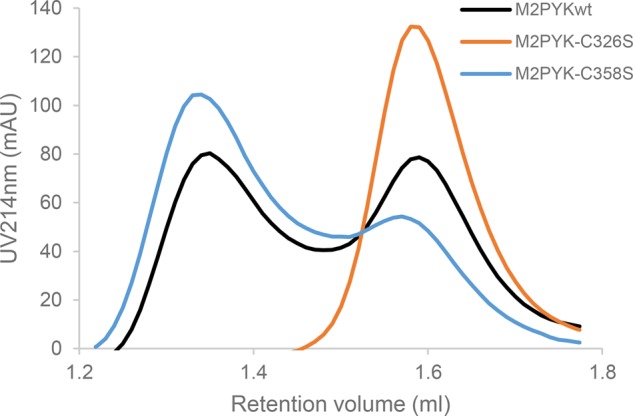
Oligomerisation states of WT M2PYK and mutants. Proteins were incubated at 0.1 mg/ml in PBS-CM at room temperature for 12 h. Analytical gel filtration was carried out to determine the tetramer (left peaks): monomer (right peaks) ratio with a Superdex® 200 PC 3.2/30 gel filtration column. Twenty-five microlitres of sample were subjected to the assay each time. Assuming a bimodal tetramer (T) : monomer (M) distribution, the peak areas correspond to WT (55% T, 45% M), C358S (79% T, 21% M), and C326S (100% M).

We have carried out analytical gel filtration of M2PYK C358S and show that the oligomerisation state of M2PYK C358S has a higher proportion of tetramer compared with WT M2PYK (Figure 9). It is, therefore, possible that the *in vivo* effect observed by Anastasiou *et al.* was due to the protection of other cysteines (e.g. Cys326 or Cys424), which are buried in the interfaces of the tetrameric PYK held stable by the C358S mutation. Analytical gel filtration of M2PYK C326S was also carried out and showed the striking result that this mutation causes M2PYK to become predominantly monomeric (Figure 9 and Supplementary Figure S1). This result supports the idea that oxidation of this C326 could play a major role in the regulation of M2PYK with the more hydrophilic sulfenic or sulfinic oxidised forms [28] favouring the monomeric form and mimicking the effect of the more hydrophilic C326S mutant. The bimodal distribution of the gel filtration traces (Figure 9) suggests that M2PYK exists predominantly as either monomer or tetramer; however, the broad saddle region between the peaks could fit with a smaller population of the dimer that may show some enzymatic activity.

**Figure 10.**
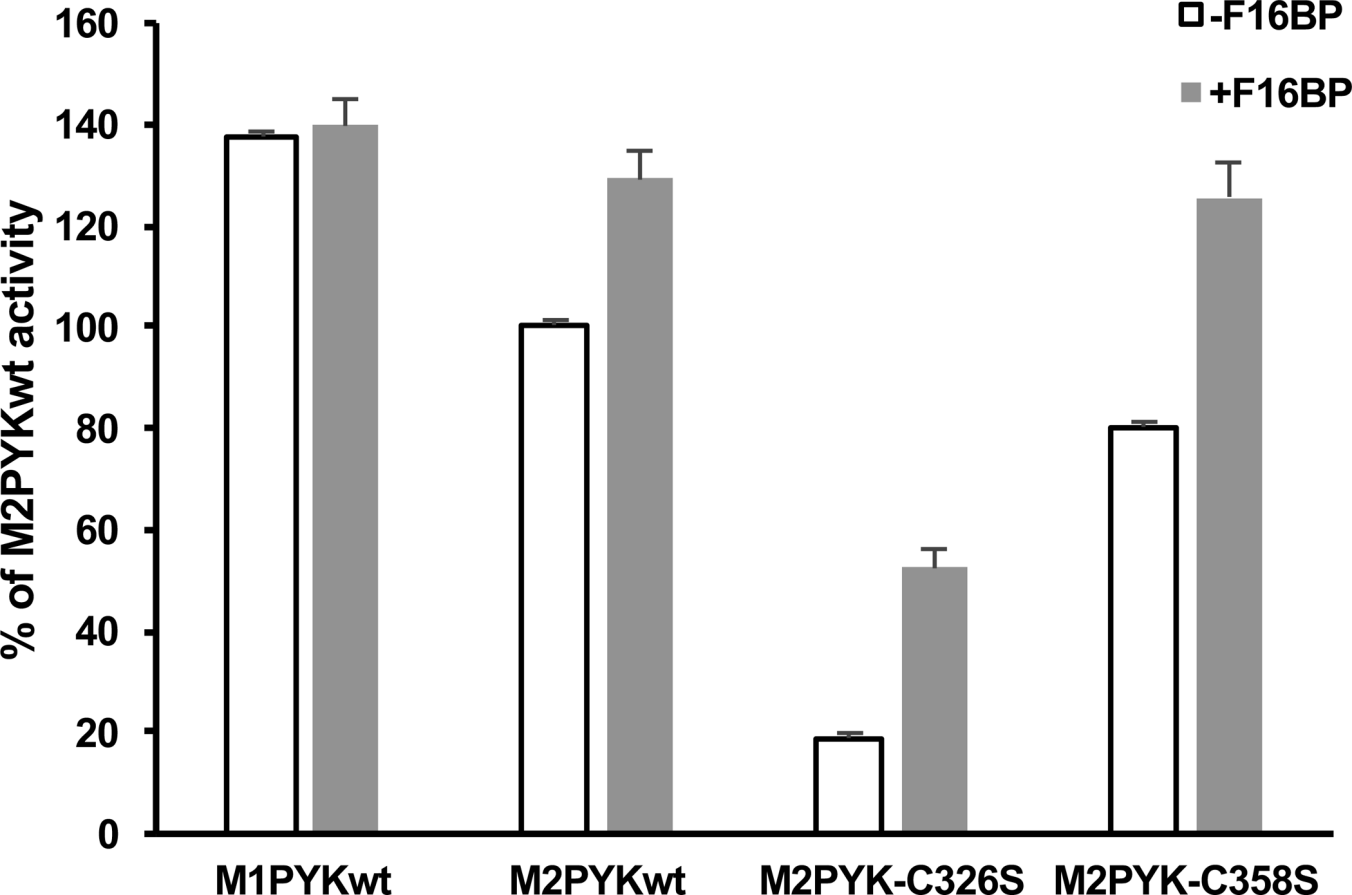
Comparisons of enzymatic activity of M1PYK and M2PYK in the absence and presence of F-1,6-BP. The activity of M2PYK C326S is much lower than that of other isoforms and mutants. It correlates with the fact that M2PYK C326S is a monomeric form of pyruvate kinase, which has a very low activity [9]. The effector F-1,6-BP activated M2PYK C326S, but only to ∼30% of the maximum activity of pyruvate kinase.

**Figure 11.**
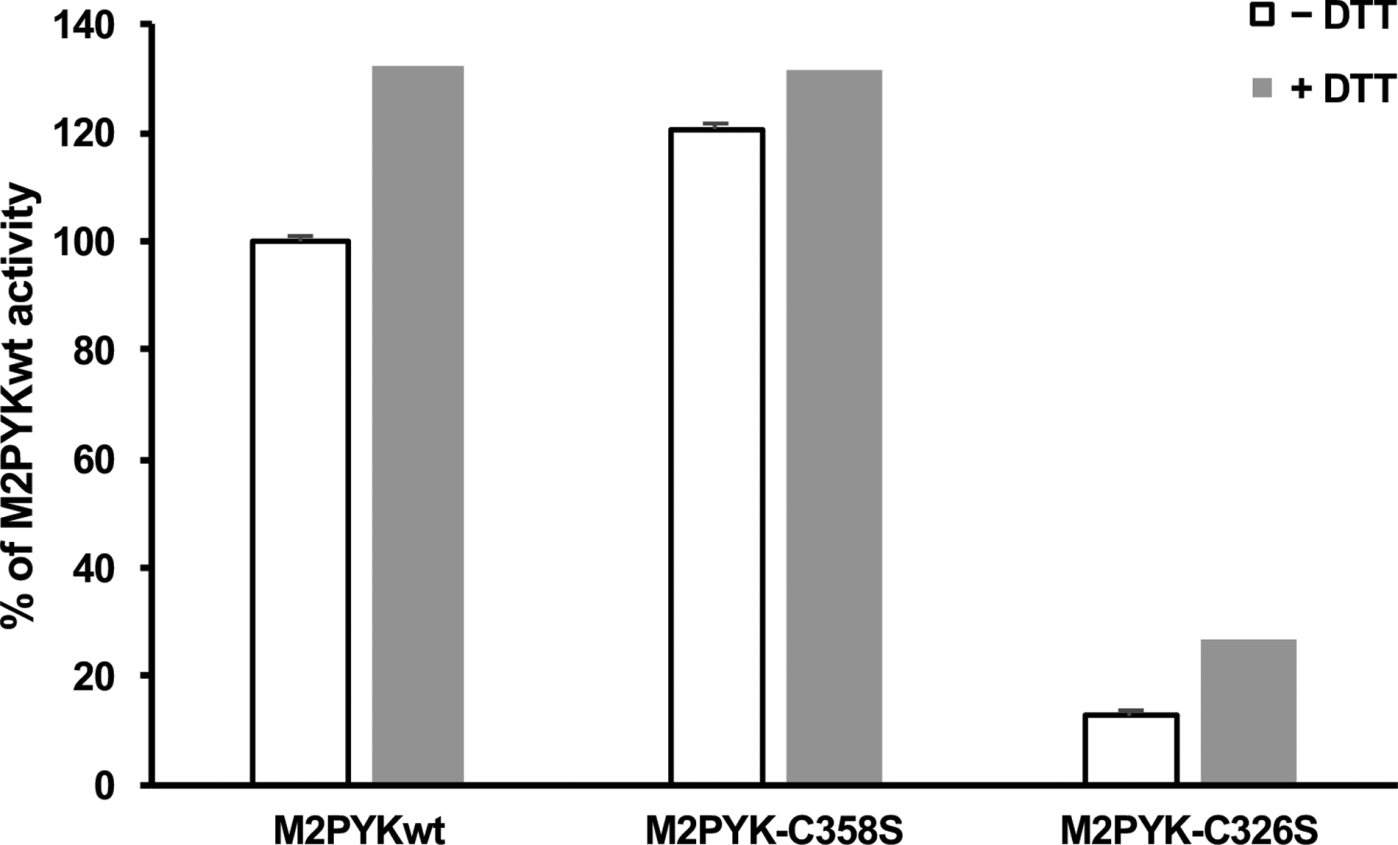
Effect of DTT on the enzymatic activity of M2PYK WT and mutants. Enzymatic activity of M2PYK, MPYK C358S and M2PYK C326S in the presence of 1 mM DTT (white) and in the absence of DTT (grey). Activities are shown relative to M2PYK in the absence of DTT (100%).

To further characterise the M2PYK C326S and C358S point mutants, their enzymatic activity was measured using the LDH-coupled assay and compared with that of M1- and M2PYK WT (Figures 10 and 11). Despite the predominantly monomeric nature of M2PYK C326S, it was found to retain ∼20% of WT activity that was increased to ∼50% with the addition of the allosteric regulator F-1,6-BP (Figure 10). The activity of M2PYK C358S was found to be very similar to that of WT both in the absence and presence of F-1,6-BP (Figure 10). The effect of the point mutations on the activity of M2PYK in oxidising (−DTT) and reducing (+DTT) conditions was also measured (Figure 11). M2PYK C358S shows some protection from oxidising conditions, having a higher activity than WT in the absence of DTT (Figure 11). The activity of the M2PYK C326S mutant is increased by the addition of DTT, suggesting that it may not be the only residue that is involved in the regulation of M2PYK by oxidation (Figure 11).

The crystal structure of M2PYK C358S was refined at a resolution of 3.1 Å. Comparison of the *B*-factors in the structure refined with cysteine in position 358 and those in the structure refined with serine in position 358 gave a clear indication that the C358S mutation had been crystallised with the serine side chain giving a good fit with the calculated electron density (Supplementary Table S2). The only other difference between the M2PYK C358S structure and M2PYK WT is the presence of Mg^2+^-ATP in the active sites of the C358S structure, which is not found in the WT structure. Interestingly, no other published human muscle PYK structures have ATP in the active site, even when crystallised with ATP present. Structural alignment of M2PYK C358S with the M2PYK WT structure (PDB ID: 3SRD) gives an overall RMS fit of 0.6 Å, indicating that there are no significant differences in backbone conformation. In the WT protein, Cys358 forms van der Waals interactions with the hydrophobic side chains of I48 and L465 which are lost in the C358S mutant.

### S-Nitrosation of M2PYK

The modification of cysteine residues by the addition of nitric oxide has emerged as an important regulatory mechanism in diverse cellular processes with over 2000 nitrosated protein targets identified, ∼580 of these protein targets being from human cells [30]. *In vivo* S-nitrosation of cysteine residues occurs via the addition of an NO moiety to a rare, highly reactive, protein cysteine thiol to form an *S*-nitrosothiol [31]. In mammals, NO is predominantly formed by nitric oxide synthase (NOS), which forms NO as a by-product of the metabolism of l-arginine to l-citrulline. There are three isoforms of NOS present in mammals; nNOS (predominating in neuronal tissue), iNOS (inducible in a wide range of cells and tissues), and eNOS (first found in vascular endothelial cells) [32]. S-nitrosation has been shown to be involved in many cancer cell phenotypes including cell growth, apoptosis, invasion, and angiogenesis [33,34].

A proteomics study using rat brain lysates has shown that only a relatively small subset of nitrosated proteins form stable nitrosothiol derivatives [35]. One of these unexpectedly stable sites was identified as rat M2PYKCys326. In a related proteomics study, in which a variety of mouse tissues were tested, PYK was found to be S-nitrosated [36]. In kidney, lung, liver, and thymus, Cys326 was identified as the only S-nitrosated cysteine in PYK, while in brain and heart nitrosated Cys326 was identified along with only one other cysteine (Cys49). The present study also showed that brain, heart and thymus M2PYK can also be nitrosated in eNOS^−/−^ mice and therefore does not require eNOS activity for nitrosation.

#### Biotin-switch assay comparing WT, C326S, and C358S M2PYK

**Figure 12.**
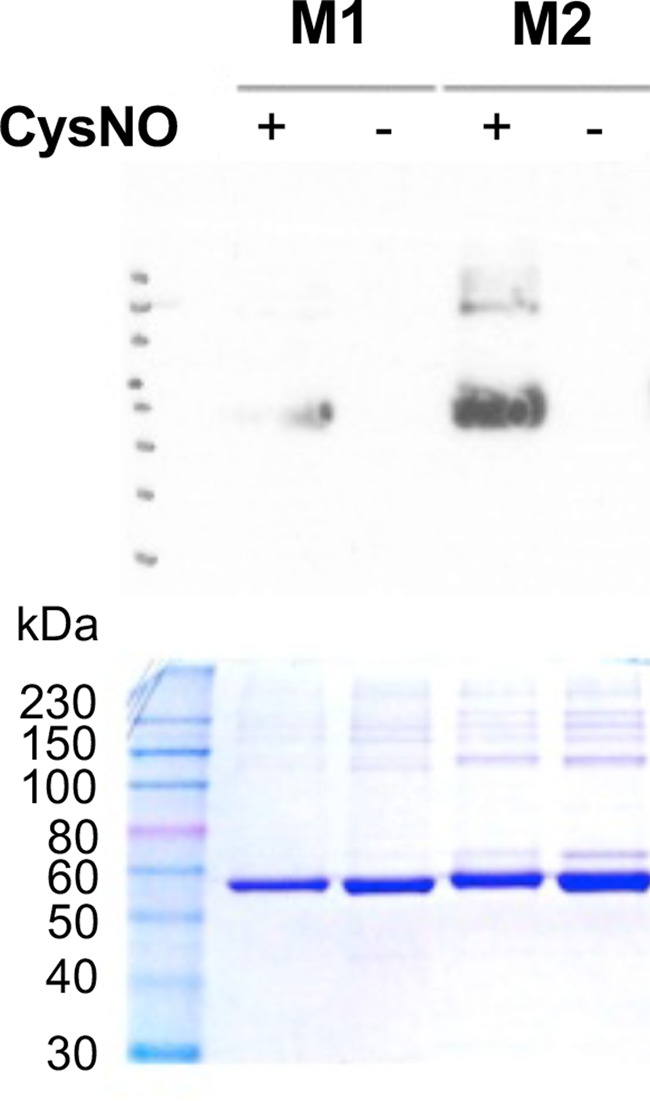
*In vitro* biotin-switch assay shows that M2PYK is more susceptible to nitrosation than M1PYK. SDS–PAGE (bottom) and anti-biotin western blot (top) of biotinylated M1PYK and M2PYK. Bacterially expressed, purified M1PYK, and M2PYK WT proteins were treated with freshly prepared CysNO (M1+ and M2+) or without CysNO (M1− and M2−). This was followed by denaturation of the protein and blocking of the unnitrosated cysteines with MMTS. Treatment with ascorbate was used to remove the nitroso moieties, which were then replaced with biotin by the addition of biotin-HPDP.

We have determined the susceptibility of human PYK to S-nitrosation using the biotin-switch technique as described in the Experimental Section [37]. Briefly, bacterially expressed, purified M1- and M2PYK WT proteins were treated with freshly prepared CysNO, followed by denaturation of the protein and blocking of the unnitrosated cysteines with methylmethanethiosulfonate (MMTS). The nitroso moieties were then removed by treatment with ascorbate and replaced with biotin by the addition of biotin-HPDP. The biotinylated proteins were then analysed by SDS–PAGE and western blot with anti-biotin antibody (Figure 12). The results show that both M1- and M2PYK are S-nitrosated, with M2PYK being S-nitrosated to a greater extent than M1PYK (Figure 12).

**Figure 13.**
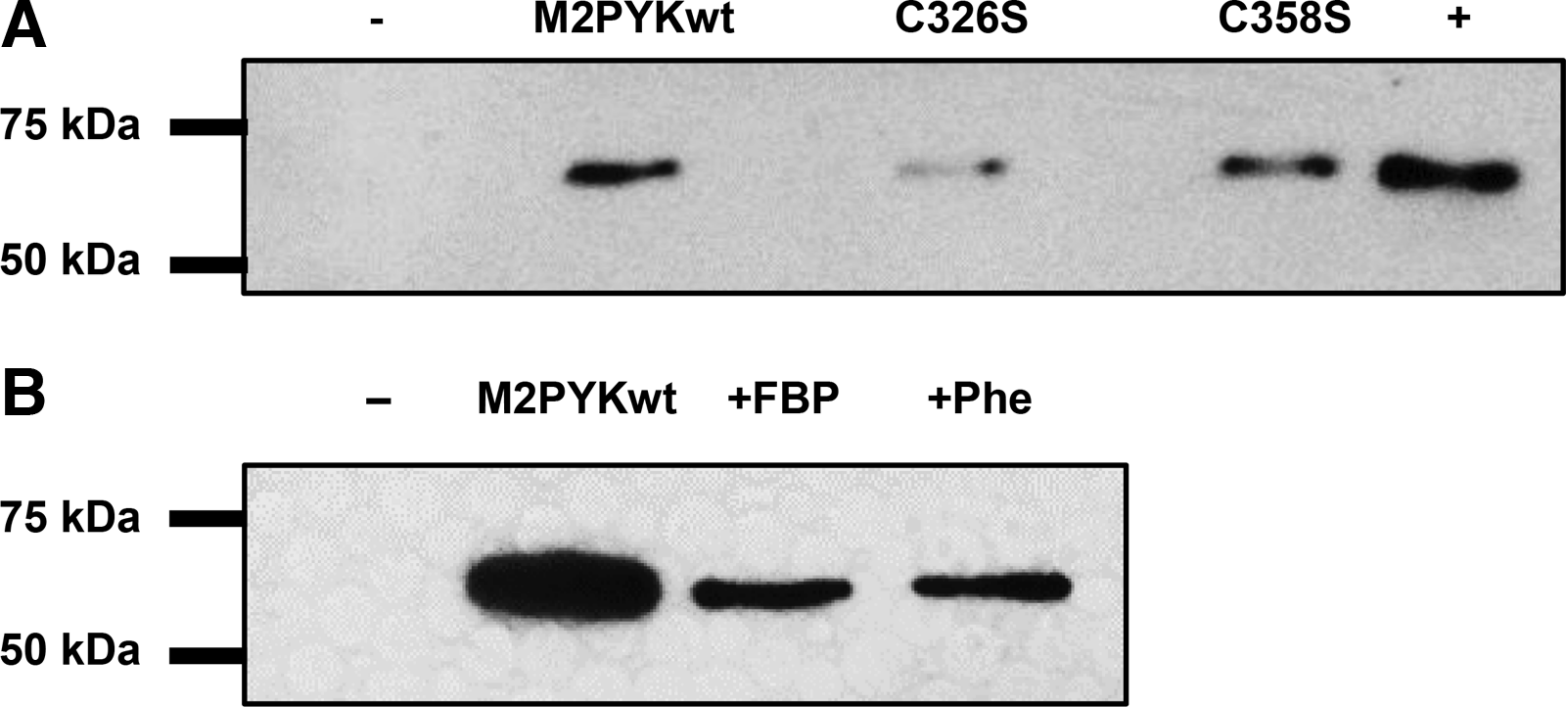
S-nitrosation of M2PYK mutants and ligand complexes. (**A**) S-nitrosation of M2PYK WT and mutants. The intensity of western blot bands reflects the degree of S-nitrosation. The negative control (−) is a protein that was not S-nitrosated. The positive control (+) is done with an unfolded protein (exposing all cysteines to S-nitrosation). The level of nitrosation is significantly reduced in the C326S mutant. (**B**) S-nitrosation M2PYK is affected by modulators. The intensity of western blot bands reflects the degree of S-nitrosation. M2PYK complexes with FBP and Phe stabilise M2PYK in a tetrameric state and reduce the amount of nitrosation. The negative control (−) is a protein that was not S-nitrosated.

To examine the role played by C326 and C358, the biotin-switch technique was carried out with M2PYK WT, and the M2PYK mutants C326S and C358S following a slightly modified protocol. The M2PYK proteins were treated with freshly prepared CysNO followed by denaturation of the protein and blocking of the unnitrosated cysteines with NEM. Ascorbate was then added to remove the nitroso moieties and the resulting free sulfhydryl groups reacted with biotin-HPDP. The biotinylated protein was then captured on streptavidin beads, eluted with β-mercaptoethanol, and analysed by SDS–PAGE and western blot with anti-His antibody. Analysis of the M2PYK C326S mutant by the biotin-switch technique shows that removing this cysteine almost completely eliminates the S-nitrosation of M2PYK (Figure 13). For comparison, the M2PYK C358S mutant was also analysed and resulted in a signal similar to the WT protein (Figure 13), showing that C326 is more prone to modification than C358.

#### Nitrosation of M2PYK is decreased when the tetrameric form is stabilised

As Cys326 is buried in the A–A interface, we hypothesised that S-nitrosation of this cysteine would be inhibited when M2PYK was stabilised in the tetrameric state. To test this, we incubated M2PYK with F-1,6-BP and phenylalanine, which have been shown to stabilise M2PYK in the tetrameric form [10]. The intensity of the western blot bands for ‘tetramer-stabilised’ M2PYK treated with Phe and F-1,6-BP shows a marked decrease, consistent with Cys326 being protected from S-nitrosation. These results are consistent with the published proteomics studies [34] and suggest that the major target of S-nitrosation in M2PYK is Cys326. A consequence of nitrosation or oxidation of this residue, which lies in the A–A interface (Figure 1), will be a shift in equilibrium towards inactive monomer formation.

## Summary and conclusion

Our results show that the difference in the effect of oxidising conditions on the enzymatic activities of M1 and M2 isoforms may be explained by the difference in their oligomeric states. M1PYK is tetrameric, whereas M2PYK is found in an equilibrium between tetramer, dimer, and monomer [9,10]. The continuous dissociation and re-association of the M2PYK monomers exposes Cys326 to oxidising conditions. Oxidation (by ROS or RNS) of Cys326 by a sulfenic or nitroso group will hinder the (re)forming of the active tetramer, thus decreasing the activity of the enzyme until the cytosol has sufficient reducing power. In contrast, the cysteines of M1PYK, which are protected within a much more stable tetramer, are less exposed to oxidising conditions.

M2PYK is a highly regulated allosteric enzyme which has already been shown to be affected by many small molecules as well as post-translational modifications such as phosphorylation, acetylation, and cysteine oxidation. We show here that oxidising conditions regulate the activity of M2PYK by pushing the oligomeric equilibrium towards the inactive monomeric form thereby reducing enzymatic activity. M2PYK has been implicated in the control of intracellular ROS that are increased in cancer cells compared with healthy cells [23]. It has been proposed that inhibition of M2PYK leads to accumulation of the glycolytic intermediate glucose 6-phosphate, which then feeds into the PPP generating the NADPH required by the enzyme GR to generate GSH for ROS detoxification [23]. Owing to the reversible nature of cysteine oxidation, once sufficient GSH has been produced to reduce the oxidised cysteines, the activity of M2PYK would be restored. In this way, M2PYK has the potential to act as a redox sensor preventing the toxic build-up of ROS or RNS in all proliferating cells, including fast growing tumours.

We also show that the effects of amino acid activators (Ser and His) and inhibitors (Ala, Phe, and Trp) are also modulated by the redox state of M2PYK adding another level of sophistication to the regulatory messages that can be processed. The term ‘allostatic regulator’ has been used to describe how many different stress and metabolic signals are combined to provide a best or most appropriate enzymatic output dependent on the current status of the cell [10]. M2PYK can sense levels from a wide range of signals including stress induced by RNS or ROS, cellular concentrations of activating metabolites (Ser, F-1,6-BP), and levels of inhibitory amino acids (Phe, Ala, and Trp). Each of these factors affects enzymatic activity by altering the conformational or oligomeric state of M2PYK. Importantly, it is not the absolute level of each of these various signals, but the relative amounts that will determine how efficiently M2PYK will produce pyruvate and ATP and provide the cell with an ‘allostatic response’ [10] to a changed cellular environment.

## Abbreviations

CVs: column volumes
DTT: dithiothreitol
F-1,6-BP: fructose 1,6-bisphosphate
GR: glutathione reductase
GSH: reduced glutathione
IPTG: isopropyl-β-d-thiogalactopyranoside
LDH: lactate dehydrogenase
LPYK: liver PYK
M2PYK: M2 isoform of human pyruvate kinase
MMTS: methylmethanethiosulfonate
NEM: *N*-ethylmaleimide
PBS-CM: phosphate-buffered saline without calcium and magnesium
PBST: PBS with 0.05% Tween 20
PEP: phosphoenolpyruvate
PPP: pentose-phosphate pathway
PYK: pyruvate kinase
ROS: reactive oxygen species
WT: wild type
