# Supplementary material for: Redox regulation of pyruvate kinase M2 by cysteine oxidation and S-nitrosation
Source: Biochem J. 2018 Oct 31;475(20):3275–91. doi: 10.1042/BCJ20180556 (PMC6208296; doi:10.1042/BCJ20180556)
Supplement: Supplementary Figures and Tables [file BCJ-475-3275-s1.pdf]

SUPPLEMENTARY

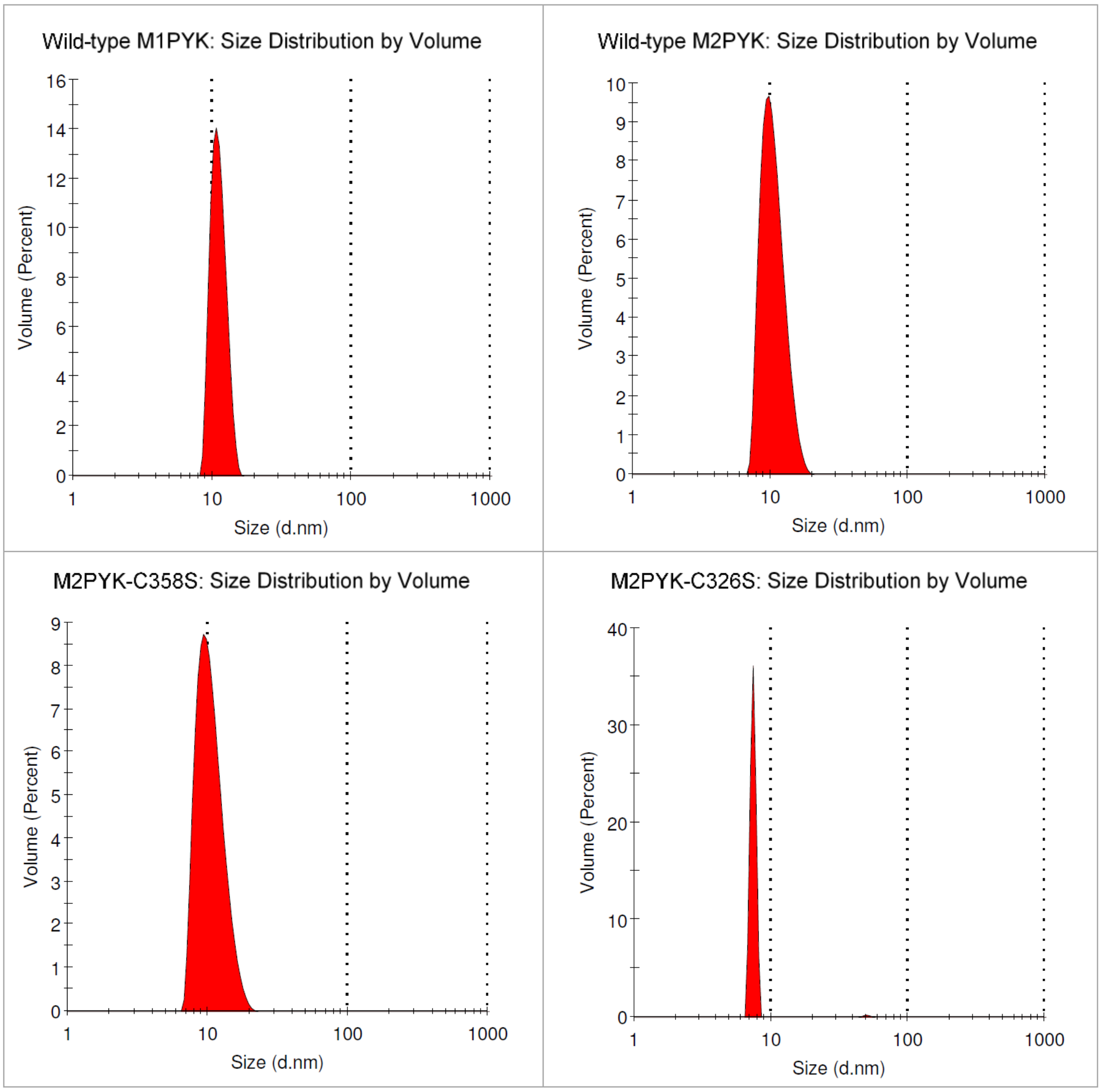

Figure S1 DLS analyses of M2PYK wild type and mutants DLS analysis of wild-type M1PYK, wild-type M2PYK and M2PYK-C358S, shows a mean hydrodynamic approximately 12 nm, respectively, for all isoforms suggesting they are predominantly tetrameric. For M2PYK-C326S, the result suggested a hydrodynamic radius of 7.4 nm, implying that M2PYK-C326S is predominantly monomeric.

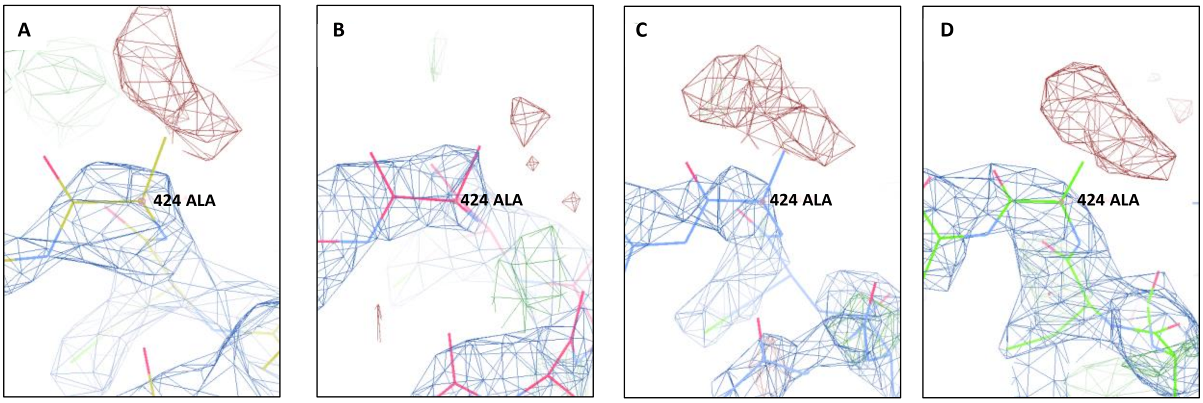

Figure S2 M2PYK C424 was successfully mutated to alanine

Electron density maps for M2PYK C424A structure showing that the cysteine was successfully mutated to alanine in all chains (A, B, C, and D). The electron density map was calculated from a model of M2PYK WT (PDB code 3SRD). The $|F_o\text{-}F_c|$ difference electron density map using the M2PYK WT model (with Cys424) shows strong negative peaks corresponding to the “missing” sulfhydryl group in the M2PYK C424A structure.

Table S1 Summary of kinetics data for PYK isoforms and cysteine point mutants

	M1PYKwt		M2PYKwt		M2PYK-C326S		M2PYK-C358S		M2PYK-C424A	
+/- F16BP	-	+	-	+	-	+	-	+	-	+
V_{max} (µmol/min/mg)	138	140	97	110	19	52	34	68	60	70
K_m[PEP] (mM)	0.13	0.15	0.9	0.17	N/D	N/D	0.9	0.13	1.3	0.18

Table S2 Comparison of B factors of atoms in residue 358 in each chain of M2PYK C358S after refinement with cysteine (red) or serine (black).

**Atom in residue**	**B factor**							
	**Chain A**		**Chain B**		**Chain C**		**Chain D**	
	**Cys358**	**Ser358**	**Cys358**	**Ser358**	**Cys358**	**Ser358**	**Cys358**	**Ser358**
**N**	79.8	75.7	90.3	84.1	77.0	77.2	48.2	45.3
**CA**	82.4	78.5	87.9	80.1	77.2	76.4	50.1	45.0
**C**	88.4	85.5	93.8	89.3	78.1	75.9	47.7	44.9
**O**	83.2	78.6	92.2	89.2	75.3	71.4	46.3	43.5
**CB**	85.1	78.1	86.7	77.0	84.4	80.9	54.0	46.5
**S/O**	103.5	81.0	111.7	83.3	95.3	78.4	82.8	54.6
